# Changes in Microstructure and Abrasion Resistance during Miller Test of Hadfield High-Manganese Cast Steel after the Formation of Vanadium Carbides in Alloy Matrix

**DOI:** 10.3390/ma15031021

**Published:** 2022-01-28

**Authors:** Grzegorz Tęcza

**Affiliations:** Department of Cast Alloys and Composites Engineering, Faculty of Foundry Engineering, AGH University of Science and Technology, 23 Reymonta Str., 30-059 Krakow, Poland; tecza@agh.edu.pl

**Keywords:** resistance to abrasive wear, Hadfield steel, high-manganese cast steel, solution treatment, microstructure, vanadium carbides

## Abstract

Hadfield cast steel is characterized by high wear resistance, but this is only when it is subjected to the effect of dynamic loads. During unloaded abrasion, e.g., sand abrasion, its wear resistance is very low and comparable to the wear of carbon cast steel. To increase the wear resistance of this alloy for operation under the conditions of low pressure or low stress, primary vanadium carbides were produced by the metallurgical process to obtain a two-phase structure after alloy solidification. Compared to samples made of Hadfield cast steel, the primary, very hard carbides, evenly distributed in an austenitic or austenitic-martensitic matrix, increase (at least three times) the wear resistance of samples tested in an abrasive mixture of silicon carbide and water. The changes in microstructure and hardness obtained in alloys after heat treatment (quenching at 1000–1150 °C in water and tempering at 600 °C) are presented. The bulk hardness of the matrix ranged from 370 HV to 660 HV. After heat treatment, the secondary, dispersed vanadium carbides, precipitated in the alloy matrix.

## 1. Introduction

**Figure 1:**
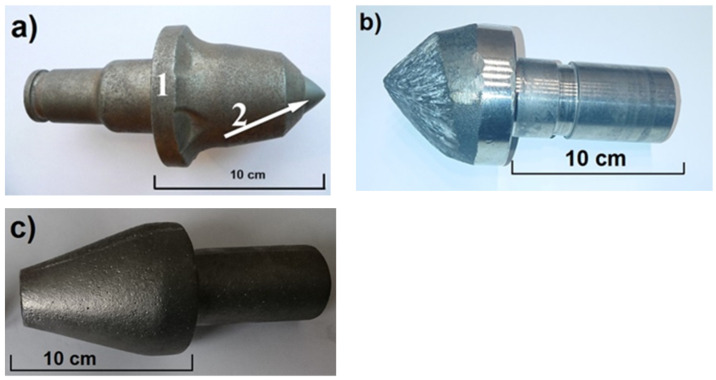
Practical example of conversion to casting technology: (**a**) forged shank, (1) part of shank made as forging, and (2) notch made by powder metallurgy; (**b**) worn out tool; and (**c**) cast shank [author].

High-manganese cast steels are characterized by high toughness, high ductility, and satisfactory wear resistance, and, therefore, they are used under service conditions that demand good resistance to impact and abrasive wear. These attributes, combined with slow crack propagation rates, afford practical benefits in rail track applications as well as in mining, construction, and rock-handling industries. High-manganese cast steels are also widely used in the power industry and the material processing industry for parts in crushers, mills, and construction machinery (linings, plates, hammers, jaws, and cones). This is mainly due to their low wear, high resistance to dynamic loads, and well preserved ductility [1,2,3]. Unfortunately, the abrasion resistance of high-manganese cast steels is only satisfactory under some specific service conditions (high strain hardening capacity as a function of high strain rate, and temperature). The low hardness of austenite (220–280 HB), depending on the chemical composition, does not guarantee the required abrasion resistance in metal–nonmetal systems. Therefore, numerous attempts have been made to improve this property, mainly by optimization of the chemical composition and introduction of new manufacturing technologies. Figure 1a illustrates this by using the example of the shank: (1) manufactured as a forged part and notch, and (2) made by powder metallurgy from WC carbides.

There are, however, some limitations in the production of each part, whether forged or cast, mainly with regard to the properties of the final product and the material qualities (under certain conditions the shank undergoes the wear and tear process more rapidly than the notch, Figure 1b). If the cast shanks contained better resistance to wear under operating conditions, the casting technology could be introduced to the industry. The use of improved grades of high-manganese cast steel with primary and secondary vanadium carbides should additionally increase the abrasion resistance, which is very important for the working conditions of the entire set, because shanks are also prone to wear during operation of the whole set of both components, Figure 1b. To achieve this purpose, a lot of attention has been paid to other processes [3,4,5] or techniques of synthesis [2,6]. The aim of the present study was to obtain the required microstructure and hardness of cast parts, and to compare their properties with those of Hadfield cast steel.

The composition of the matrix of austenitic manganese cast steels varies, and it is selected in such a way as to obtain the optimal combination of wear resistance, ductility, and strength. The standard grades (Table 1) are used for heavy crusher castings, railroad components, and teeth or other elements used in machinery industry [1,7,8,9].

Most high-manganese grades (Table 1) are characterized by a high degree of strain-hardening due to the transformation of austenite into ε—martensite or α’—martensite, or due to the occurrence of mechanical twinnings [6,7,10,11,12,13,14,15,16,17,18,19,20]. However, current research and development activities mainly focus on dual-phase alloys [2,4,6], including high-manganese cast steels, whose matrix is reinforced with carbides and nitrides [2,4,5]. Castings for industrial applications become parts of machines that need to be replaced, even if they only suffer a loss of a few millimeters. Hence, the tendency evolves to keep the casting core ductile, while hardening only the surface of the cast element. This is achieved in various ways, e.g., the surface of a high-manganese steel casting is subjected to explosive hardening [21], and, recently, similar to tool steels, vanadium carbides are introduced during metallurgical process or by using SHS powder synthesis (Self-propagating High-temperature Synthesis). In the latter case, carbides are formed in liquid alloy as a result of the reaction proceeding under the effect of the high temperature created in the alloy and carbides synthesis from the mixture of powders.

**Table 1:** Chemical composition of Hadfield cast steels for operation under the conditions of wear and work-hardening.

Designations	Chemical Composition [wt%]									Refs.
	C	Mn	Si	P	S	Cr	Ni	Mo/W	Ti/V	
**Conventional grades**	1–1.4	12–14	0.3–1	<0.10	<0.03	<1.0	<1.0	-	-	[1]
	1–1.4	12–14	0.3–1.0	<0.1	<0.03	0.6–1.3	<0.5	-	-	[1]
	1.05–1.35	11.5–14	<1.0	0.07	-	1.5–2.5	-	-	-	[7]
	0.7–1.3	11.5–14	<1.0	<0.07	-	-	3–4	-	-	[1,9]
	1.05–1.45	11.5–14	<1.0	<0.07	-	-	-	1.8–2.1	-	[1,9]
	1.05–1.35	6–8	<1.0	<0.07	-	-	-	0.9–2.1	-	[1,9]
	0.9–1.5	10–14	-	-	-	1–2	-	<2	-	[4]
	1.40	20.02	0.44	0.074	0.007	2.50	0.13		Ti = 0.02	[6]
**Reinforced**	0.9–1.5	10–14	-	-	-	1–2	-	<2	-	[4]
	1.2	15	1.5	0.04	0.02	1.4	0.1	-	Ti = 2.5	[16,17]
	2.72	17.10	0.45	0.08	0.02	0.43	-	-	Ti = 10.0	[5]
	2.96	16.86	0.45	0.05	0.03	0.34	-	W = 3.0	Ti = 3.02	[5]
	2.60	14.30	0.75	0.031	0.014	1.41	-	0.07	V = 8.1	[15]

Modern technology is largely based on these methods, allowing for the production of castings with composite zones resistant to abrasion, while maintaining a plastic core [22,23].

The search for new techniques and technologies for tool production prompted the author to develop a concept that would meet the assumption that tool steels and cast steels can be expected to offer high abrasion resistance, as well as plasticity and crack resistance, at notably elevated temperatures. These properties are due to the morphology of MC and M_2_C carbides that occurs in an austenitic alloy matrix. However, the presence of “coarse” (thick) MC carbides in as-cast state makes the ductility of cast steel drop quite significantly [18,19].

In their unpublished studies, Głownia et al. [20] have shown that, in the cast tool steels with vanadium carbides (VC) used so far, eutectic is the preferred type of morphology, because the content of primary carbides is up to 20%. This concept accompanied the introduction of an increased amount of vanadium (up to 12%) and carbon (2.3–2.8%) into high-manganese steel, producing a hardness of 800 HV, mainly due to the appearance of martensite, and to the presence of primary and secondary vanadium carbides. Using abrasion tests, they have also demonstrated that the tested cast steel, containing from 7% to 12% of vanadium, occupies a position close to present day high-speed tool steels [10].

The satisfactory results of studies measuring the change in microstructure and abrasion resistance of high-manganese cast steel with the addition of Ti and Nb, obtained thus far in the Miller test [15,16,24], have prompted the author to conduct research on high-manganese cast steel with the addition of vanadium.

The studies presented in [24] are a review and comparison of the author’s past research on the formation of primary and secondary carbides in high-manganese cast steel in the metallurgical process, and, subsequently, on the related changes in abrasion resistance.

## 2. Materials and Methods

The test specimens were cut out from the “Y”-type ingots with a wall thickness of 25 mm and a weight of about 1 kg, cast from the steel melted in a 100 kg capacity induction furnace when making pilot castings of crusher teeth. The furnace charge was Hadfield cast steel scrap of known chemical composition (increased Cr content). After melting the charge into liquid steel, to supplement the chemical composition, if necessary, a carburizer in the form of pig iron of known chemical composition was added in an appropriate amount during the metallurgical process along with ferroalloys such as Fe-Mn65, Fe-Si65, and Fe-V80. Adding vanadium in the final stage of the melting process triggered the formation of primary vanadium carbides in liquid steel, evenly distributed in the alloy matrix after casting solidification.

**Figure 2:**
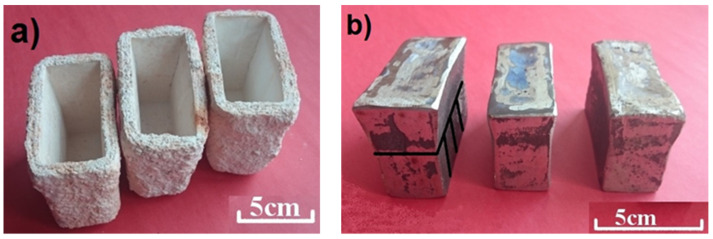
(**a**) molds used in tests. (**b**) examples of “Y” type test castings made from the examined steel and scheme of their cutting.

To reduce the oxidation process of the alloying additives to minimum, the melt was deoxidized in two stages [25], i.e., with Al (added in the amount of 1 kg/1 Mg of steel) in the furnace shortly after melting the charge and before adding vanadium, and then with Fe-Ca-Si (added in the amount of 1 kg/1 Mg of steel) during tapping into the ladle. The pouring temperature was 1560–1570 °C, and the steel was poured into ceramic molds obtained by the lost-wax technology. Figure 2a shows molds ready for pouring with molten alloys, while Figure 2b shows the obtained castings.

After solidification and cooling (next day), the castings were cleaned and subjected to heat treatment which consisted of solution treatment at a temperature from 1000 °C to 1150 °C with a step of 50 °C, holding at a given temperature for 0.5 h and cooling in water. The castings were next tempered at a temperature of 600 °C for 2 h and cooled in air. Taken from castings after heat treatment, samples were cut out for structural examinations and abrasion resistance tests. The scheme of cutting out the samples is shown in Figure 2b alongside the scheme of cutting them through.

Chemical analysis of the tested alloys was carried out under industrial conditions using a Foundry Master spectrometer and was then completed with laboratory examinations using an energy dispersive X-ray fluorescence spectrometer. Table 2 shows the chemical composition of the tested alloys.

Chemical analysis of the composition of test ingots has shown that, compared to the conventional GX120Mn13 cast steel, the cast steel melted in the tests was characterized by an increased content of carbon (1.7% to 2.6%), increased content of silicon (0.7% to 1.9%), which was the result of double deoxidation with Fe-Ca-Si and Al alloys, and increased content of chromium (from 1.4% to 1.7%), which was the result of chromium content in the charge. In the tested alloys, a different manganese content was obtained (from 9.8% to 14.3%), and it was increasing with the carbon content in the alloy. The content of vanadium also varied, amounting to 5.5%, 6.3%, and 8.1%.

The microstructures of the tested alloys were examined under a Neophot 32 (Carl Zeiss Jena, Hövelhof, Germany) light microscope equipped with a camera for digital image recording. Chemical analysis of the composition of carbides present in the tested alloys and of the alloy matrix was performed with a FEI SEM XL30 (FEI Company, Hillsboro, OR, USA) scanning electron microscope equipped with an EDAX GEMINI 4000 (FEI Company, Hillsboro, OR, USA) energy-dispersive X-ray spectrometer.

Phases present in the tested samples were identified with a Kristalloflex 4H (Siemens, Munich, Germany) X-ray diffractometer from Siemens using the characteristic Cu radiation (Kα = 0.154 nm) with a step size of 0.052 theta/1 s.

**Figure 3:**
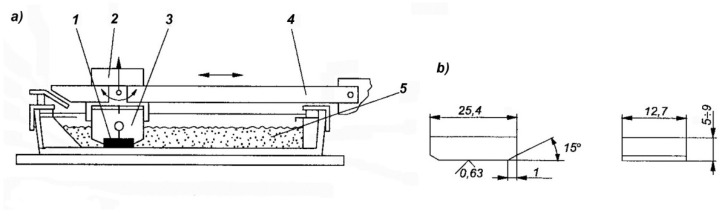
Diagram of the machine used in the Miller test. (**a**): 1, specimen, 2, weight, 3, specimen holder, 4, holder arm, and 5, abrasive. (**b**) dimensions of the specimen tested for abrasive wear.

The abrasive wear response was determined in the Miller test conforming to ASTM G75, which is used to compare the abrasive wear behavior of various construction materials. Figure 3 shows a diagram of the machine used in the Miller test.

This method allowed the author to compare the previously obtained results with the results obtained by other members of the research team [14,16,24]. The test specimens, with dimensions of 25.4 × 12.7 mm and a thickness of 9 mm, were placed in the holders of the device under a constant load of 22.2N and were then subjected to abrasion in a mixture of water and silicon carbide in a 1:1 ratio. The counter-sample was the rubber lining of the trough bottom where the abrasion process took place. Silicon carbide with grain number 220 according to FEPA standard and grain size 53–73 µm was used. Two 16-h abrasion tests were performed in 4 cycles for each sample, calculating the mean. Every four hours, the sample was weighed with an accuracy of 0.001 g. Based on the obtained values of mass losses, abrasive wear curves were plotted for the tested samples. The values of wear obtained for the samples of the tested alloys were compared with the values of wear obtained for the reference sample made of Hadfield cast steel with 1.2% C, 13% Mn, and 0.8% Si, which was subjected to standard solution heat treatment, from the temperature of 1050 °C, and whose hardness was 230 HV.

The surface of the samples after abrasive wear tests was macroscopically compared with the surface of the Hadfield cast steel sample.

Further analysis of the surface condition requires profilometric tests, which are a standard method used for the assessment of surface condition after various technological operations. These tests will be carried out by the author in further research on the presented alloys.

## 3. Test Results and Discussion

In this study, various issues, related to the microstructural characterization, hardness, and abrasive wear resistance of austenitic and austenitic-martensitic high-manganese cast steels with vanadium carbides, are discussed.

**Figure 4:**
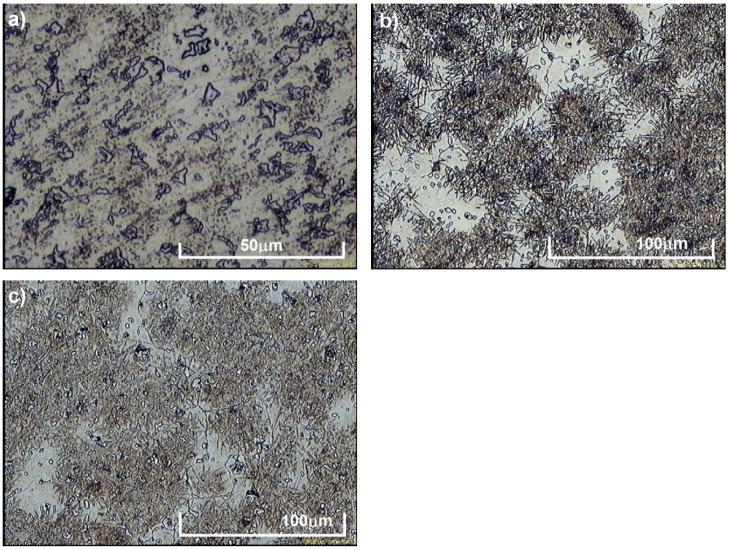
As-cast microstructure produced in test castings containing: (**a**) 5.5% V; (**b**) 6.3% V; and (**c**) 8.1% V, etching with nital.

Figure 4 shows the microstructure of test castings in as-cast state. Based on the examinations carried out by light microscopy, it was found that the microstructure of the tested alloys with a vanadium content of 5.5% consisted of faceted vanadium carbides evenly distributed in an austenitic matrix (Figure 4a). With respect to vanadium contents of 6.3% and 8.1%, the structure of the tested alloys was composed of a martensitic-austenitic matrix with primary and eutectic vanadium carbides (Figure 4b,c). The martensitic-austenitic structure produced in as-cast state proves the high hardenability of the obtained alloys. The as-cast hardness of the tested alloys increases with carbon content and vanadium addition (Table 3) and ranges from 411 HV to 547 HV.

**Figure 5:**
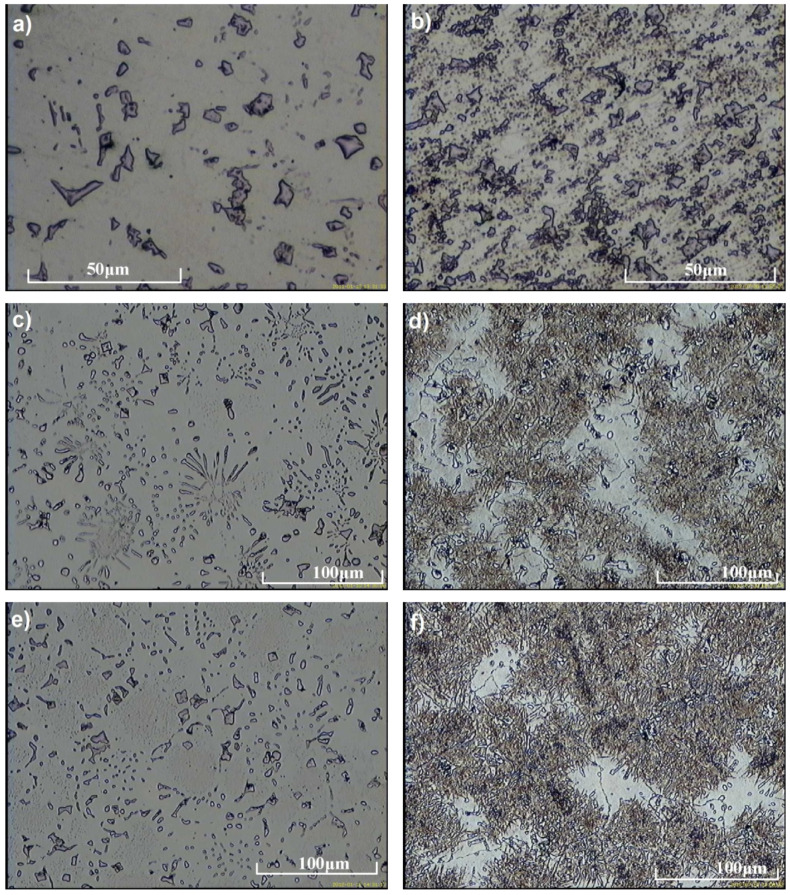
Microstructure of the tested alloys containing: (**a**,**b**) 5.5% V; (**c**,**d**) 6.3% V; and (**e**,**f**) 8.1% V after heat treatment. (**a**,**c**,**e**) unetched specimens; primary and eutectic vanadium carbides evenly distributed in alloy matrix. (**b**,**d**,**f**) specimens etched with nital; bright austenite, dark martensite, primary and eutectic vanadium carbides evenly distributed in alloy matrix.

Table 3 presents the heat treatment parameters and the average hardness values obtained for the tested alloys after heat treatment. The hardness obtained after solution treatment and tempering increases with carbon content, vanadium addition, and temperature of solution treatment (Table 3). The increase is due to the presence of martensite in the alloy matrix (Figure 5) and the release of secondary dispersion vanadium carbides (Figure 8). The highest hardness (660 HV) was obtained for the alloy containing 8.1%V after solution treatment from the temperature of 1000–1100 °C, with cooling in water, and tempering for 2 h. For this vanadium content (8.1%), the temperature of solution treatment had no effect on the hardness obtained after heat treatment.

**Figure 6:**
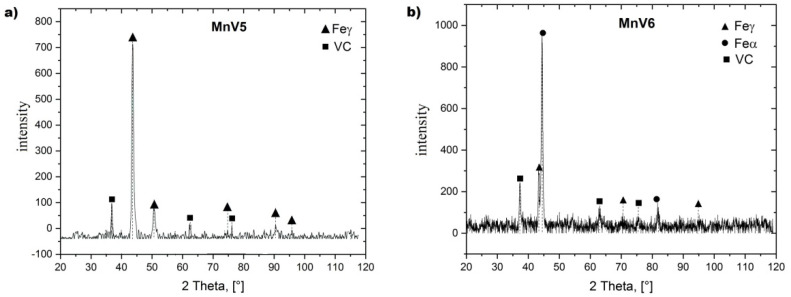
X-ray diffraction patterns of alloys containing (**a**) 5.5% V, and (**b**) 6.3% V.

**Figure 7:**
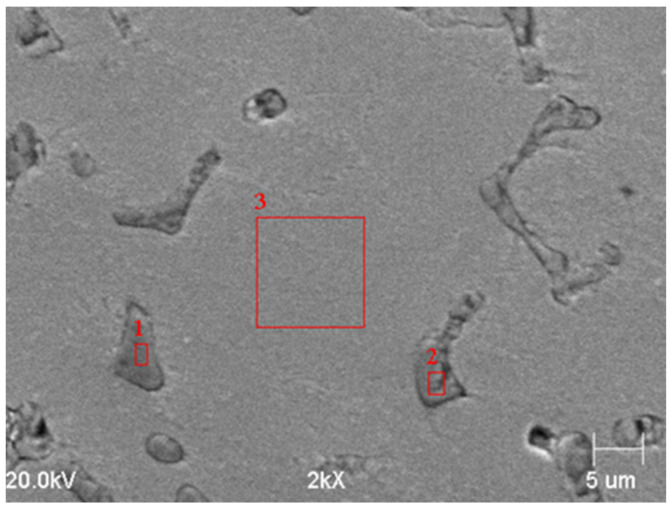
SEM image of an alloy containing 5.5% V after solution treatment; austenitic matrix with primary vanadium carbides; the red marks denote places of analysis.

**Figure 8:**
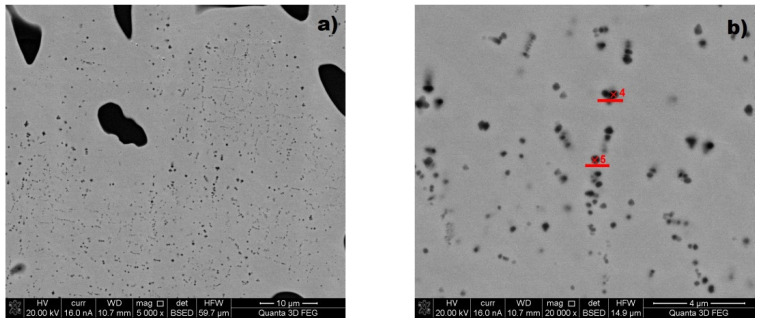
(**a**) examples of secondary carbides visible in an alloy containing 6.3% V. (**b**) red marks denote places of analysis for the alloy containing 8.1% V.

Figure 5 shows the microstructure of the tested alloys after the performed heat treatment, while Figure 6 shows the X-ray diffraction patterns of the tested alloys. Figure 7 and Figure 8 show examples of SEM images with analysis of the visible particles in Table 4 and Table 5.

Based on the observations carried out, it was found that, after solution treatment, the microstructure of the tested cast steel, with vanadium content of 5.5%, was composed of an austenitic matrix with vanadium carbides evenly distributed in this matrix (Figure 5a,b, Figure 6a, and Figure 7). With the increase in vanadium content to 6.3% and 8.1%, martensite appeared in the microstructure (Figure 5d,f, and Figure 6b). The morphology of vanadium carbides also changed. For the 5.5% V content, the carbides were of a faceted type (Figure 5a), while for higher V contents they were mostly eutectic (Figure 5c,e).

In the samples with 6.3% and 8.1% V after solution treatment and tempering, the SEM images have revealed the presence of the secondary precipitates of dispersion vanadium carbides evenly distributed in the alloy matrix (Figure 8). Due to their small size, the analysis performed was inaccurate, and it was undertaken only for the alloy matrix, but the precipitates were vanadium-rich particles. Table 5 shows an approximate chemical composition of the visible precipitates.

### Wear Resistance

**Figure 9:**
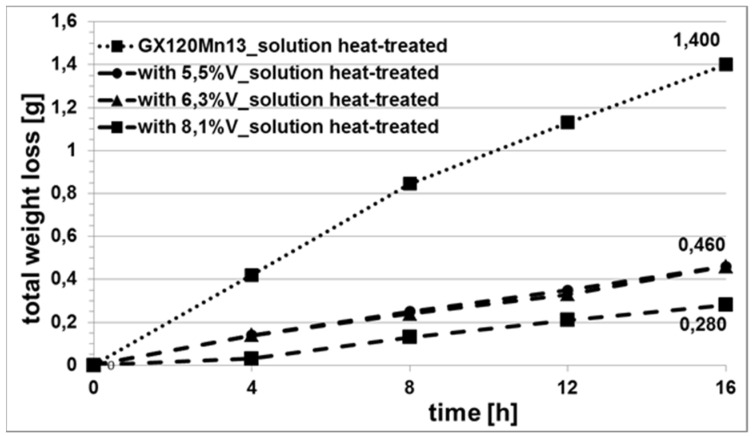
Total weight losses of the tested alloys as a function of abrasion time.

The abrasive wear resistance tests were carried out on the samples which were solution-treated from the temperature of 1050 °C, because this type of heat treatment is recommended as a standard procedure for Hadfield cast steel. Figure 9 shows the total weight losses of samples plotted as a function of abrasion time.

Based on the obtained results of the weight losses of the tested samples, it was found that, compared to conventional Hadfield cast steel, the abrasive wear resistance of the tested high-manganese cast steel, with the addition of 5.5% and 6.3% V, increased over three times (from 1.40 g/16 h to 0.460 g/16 h). Increasing the content of vanadium to 8.1% further reduced the sample wear, and, amounting to 0.280 g after 16 h, the abrasive wear was five times lower. The obtained results can be compared with the abrasive wear of high-manganese cast steel containing the addition of niobium [24], which was tested for abrasive wear under identical conditions. The addition of 3.5% Nb to the alloy brought an identical reduction in wear, i.e., from 1.4 g/16 h to 0.461 g/16 h, and, with the Nb content raised to 4.5%, the wear rate was 0.366 g/16 h, which increases the abrasion resistance nearly four-fold. These results lead to the conclusion that one of the factors determining the rate of wear is the amount of carbides produced in the alloy matrix.

**Figure 10:**
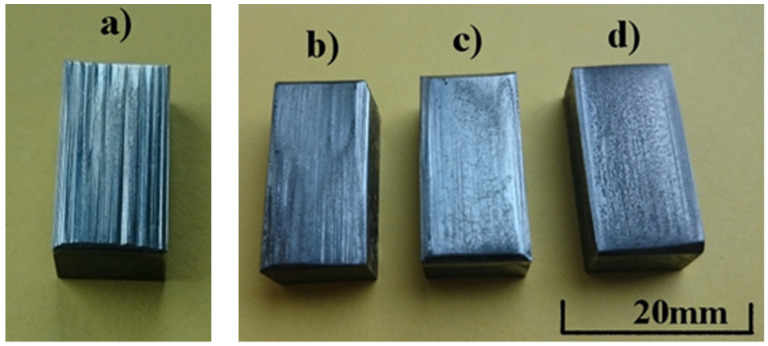
Surface of the samples after the abrasion test: (**a**) reference sample made of GX120Mn13 cast steel; (**b**) high-manganese cast steel with the addition of 5.5% V; (**c**) high-manganese cast steel with the addition of 6.3% V; and (**d**) high-manganese cast steel with the addition of 8.1% V.

Figure 10 shows macroscopic images of the surface of the samples after the abrasion test compared with the reference sample made of Hadfield cast steel.

The abrasive wear of the sample made of Hadfield cast steel is very uneven: deep scratches and furrows are visible on the surface (Figure 10a). The addition of vanadium makes this wear more uniform. With the increasing content of vanadium, the furrows and scratches are smaller, and the surface of the samples becomes “smooth” (Figure 10b,c). The wear of the sample surface is most uniform when the content of vanadium reaches 8.1% V. The surface is rough and rugged in a uniform way with vanadium carbides protruding from the alloy matrix (Figure 10d). Further analysis of the surface condition and wear mechanism requires separate tests, and these will be conducted as part of the next research program.

## 4. Conclusions

Primary and secondary carbides were produced by the conventional melting, casting (solidification), and heat treatment route. These particles improve both the hardness and abrasive wear resistance of new Hadfield grades. Based on the present investigations, it can be concluded that high-manganese cast steels, reinforced with vanadium carbides, have considerable potential for industrial application. However, the obtained values of hardness are still not up to par with the expected results. Therefore, the next investigations must involve higher contents of vanadium, alongside a careful adjustment of the carbon content, to match these values. Heat treatment, especially the temperature of tempering, will play an equally important role.

From this study, the following conclusions can be drawn:

1. With vanadium content of 5.5%, the microstructure consists of faceted vanadium carbides evenly distributed in an austenitic matrix.
2. For vanadium contents of 6.3% and 8.1%, the structure of the tested alloys is composed of a martensitic-austenitic matrix with primary and eutectic vanadium carbides.
3. The as-cast hardness of the tested alloys increases with carbon content and vanadium addition, ranging from 411 HV to 547 HV.
4. The highest hardness (660 HV) was obtained for the alloy containing 8.1% V after solution treatment from the temperature of 1000–1100 °C, with cooling in water, and tempering for 2 h.
5. The abrasive wear resistance of the tested high-manganese cast steel with vanadium addition is at least three times higher than that of Hadfield cast steel.
6. Owing to the addition of vanadium, the wear of the samples is more uniform without furrows and scratches.

## Figures and Tables

**Table 2:** Chemical composition of the tested high-manganese cast steel.

Designations	Chemical Composition [wt%]									
	C	Mn	Si	P	S	Cr	Ni	Mo	Al	V
MnV5	1.65	9.80	1.94	0.038	0.013	1.66	0.3	0.05	0.03	5.5
MnV6	2.35	12.1	0.72	0.033	0.016	1.40	0.2	0.07	0.04	6.3
MnV8	2.60	14.3	0.75	0.031	0.014	1.41	0.3	0.07	0.03	8.1

**Table 3:** Changes in hardness values as a function of vanadium content and temperature of quenching and tempering.

Designations	Average Hardness HV				
	As-Cast	Q:1000 °C/w	Q:1050 °C/w	Q:1100 °C/w	Q:1150 °C/w
		Tempering at 600 °C/2 h/air			
MnV5	411	370	365	410	440
MnV6	515	600	635	635	615
MnV8	547	660	660	660	600

**Table 4:** Sample chemical compositions of the visible precipitates (Figure 7).

Spot	[wt%]					
	C	V	Mn	Fe	Si	Ti
Point 1	rest	49.4	-	-	0.7	0.7
Point 2	rest	45.2	-	-	0.6	-
Point 3	rest	3.7	8.2	86.4	1.6	-

**Table 5:** Sample chemical compositions of the visible precipitates (Figure 8).

Spot	[wt%]					
	C	V	Mn	Fe	Si	Cr
Point 4	rest	21.1	6.2	54.5	0.8	0.9
Point 5	rest	24.3	5.1	52.1	0.7	0.8

## References

[1] Wieser P.R. (1980). Wear-Resistant Steels. Steel Castings Handbook.

[2] Pagounis E., Lindroos V.K. (1998). Processing and properties of particulate reinforced steel matrix composites. Mater. Sci. Eng. A.

[3] Takeda M., Mitome M., Hayakawa H., Nishiuchi S., Tanabe T., Yamamoto S. (2013). Morphology and Crystallographic Phase of V-C Particles Formed in Fe-Cr-Ni-V-C Alloys. Mater. Sci. Technol..

[4] Xie P.J., Zhang C.T., Wang W.Y., Wang A.Q. Study on the Microstructure of High Manganese Steel Composites Strengthened by Ceramic. Proceedings of the Cast Steels and Superalloys, 69th World Foundry Congress.

[5] Srivastava A.K., Das K. (2009). Microstructural and Mechanical Characterization of in Situ TiC and (Ti,W)C-Reinforced High Manganese Austenitic Steel Matrix Composites. Mater. Sci. Eng. A.

[6] Das K., Bandyopadhyay T.K., Das S. (2002). A review on the various synthesis routes of TiC reinforced ferrous based composites. J. Mater. Sci..

[7] Kattamis T.Z., Suganuma T. (1990). Solidification Processing and Tribological Behavior of Particulate TiC-Ferrous Matrix Composites. Mater. Sci. Eng. A.

[8] (2015). Austenitic Manganese Steel Castings.

[9] Krawiarz J., Magalas L. (2005). Modified cast Hadfield steel with increased wear resistance. Foundry J. Pol. Foundrymen’s Assoc..

[10] Cina B. (1954). Transformation →αFe-Cr-Ni Alloys.

[11] Troiano R., McGuive F.T. (1943). A study of the iron-rich iron-manganese alloys. Trans. ASM.

[12] Bouaziz O., Allain S., Scott C.P., Cugy P., Barbier D. (2011). High manganese austenitic twinning induced plasticity steels: A review of the microstructure properties relationships. Curr. Opin. Solid State Mater. Sci..

[13] Roberts W.N. (1964). Deformation twinning in Hadfield steel. Trans AIME.

[14] Głownia J., Tęcza G., Asłanowicz M., Ościłowski A. (2013). Tools cast from the steel of composite structure. Arch. Metall. Mater..

[15] Tęcza G., Garbacz-Klempka A. (2016). Microstructure of cast high-manganese steel containing titanium. Arch. Foundry Eng..

[16] Tęcza G., Zapała R. (2018). Changes in impact strength and abrasive wear resistance of cast high manganese steel due to the formation of primary titanium carbides. Arch. Foundry Eng..

[17] Karagoz S., Yilmaz A. Cast high speed tool steels with niobium addition. Proceedings of the 66th Word Foundry Congress, Proceedings: Casting Technology 5000 Years.

[18] Haberling E., Rasche K., Wendl F., Thyssen K.D. (1993). Fortschrichtte und Entwicklungstendenzen auf dem Gebiet der Werkzeugst ähle. Thyssen Tech. Ber..

[19] Villars P., Prince A., Okamoto H. (1995). Handbook of Ternary Alloy Phase Diagrams, 5.

[20] Głownia J., Krawiarz J. (1990).

[21] Stradomski Z. (2001). Quantitative analysis of stacking faults and microtwins in Hadfield cast steel strengthened by the explosion method. Analiza ilościowa błędów ułożenia i mikrobliźniaków w wybuchowo umacnianym staliwie Hadfielda. Arch. Nauk. O Mater..

[22] Olejnik E., Janas A., Kolbus A., Sikora G. (2011). The composition of reaction substrates for TiC carbides synthesis and its influence on the thickness of iron casting composite layer. Arch. Foundry Eng..

[23] Olejnik E., Tokarski T., Sikora G., Sobula S., Maziarz W., Szymański Ł., Grabowska B. (2018). The Effect of Fe Addition on Fragmentation Phenomena, Macrostructure, Microstructure, and Hardness of TiC-Fe Local Reinforcements Fabricated In Situ in Steel Casting. Metall. Mater. Trans. A.

[24] Tęcza G. (2021). Changes in abrasive wear resistance during Miller test of high-manganese cast steel with niobium carbides formed in the alloy matrix. Appl. Sci..

[25] Kalandyk B., Zapała R., Sobula S., Tęcza G. (2019). The effect of CaSiAl modification on the non-metallic inclusions and mechanical properties of low-carbon microalloyed cast steel. Arch. Foundry Eng..

